# Medical Department University of Buffalo

**Published:** 1855-09

**Authors:** 


*Medical Department University of Buffalo.*—With reference to the convenience of *Physicians*, many of whom, it is believed, are desirous of reviewing their studies, and of becoming familiar with the progress of their profession, the Faculty of the University of Buffalo have set apart the month of January for Lectures upon subjects especially interesting and important to the medical practitioner. The Lectures constitute a portion of the regular course, but will be delivered, as selected, and at the least busy season of the year, in order to accommodate those who cannot, for a longer time or at another period, be spared from the active duties of their profession.

This course is *free* to all duly authorized practitioners, upon the payment of the matriculation fee, ($5,) for which a ticket will be given, admitting the holder to all the Lectures at the College, as also to the medical and surgical clinics at the Hospital.

Circulars will be issued before the course commences, announcing the subjects to be taught, which will be selected with especial reference to the wants of practitioners. By thus condensing within a month those matters of greater practical interest, ordinarily scattered through the entire term, physicians will be enabled to refresh themselves on important subjects, without being obliged to listen to those elementary lectures which are valuable only to students.

It is the wish of the Faculty to establish the most intimate relations between themselves and the profession at large, and we know of no way so likely to secure this result, as the personal acquaintance — profitable, we trust, to both parties — which would follow the collection of a large number of physicians in Buffalo next January. The unrivalled clinical advantages of the Buffalo school — indisputably better than those of any other city in the Union — will add great interest to the contemplated course.

